# Association between perceived stigma and satisfaction with diabetes technologies among adolescents and young adults with type 1 diabetes: a cross-sectional study in Turkey

**DOI:** 10.7717/peerj.20884

**Published:** 2026-03-05

**Authors:** Gülistan Çoban, Hamdiye Arda Sürücü

**Affiliations:** 1 Internal Medicine Nursing, Faculty of Health Sciences, Bingöl University, Bingöl, Turkey; 2 Internal Medicine Nursing, Atatürk Faculty of Health Sciences, Dicle University, Diyarbakır, Turkey

**Keywords:** Diabetes, Type 1 diabetes, Stigma, Diabetes technologies, Satisfaction

## Abstract

**Background/Aim:**

There was no clinical evidence regarding the relationship between stigmatization and satisfaction with diabetes technologies in persons with type 1 diabetes. The aim of this cross-sectional study is to examine the association between perceived stigma and satisfaction with diabetes technologies among adolescents and young adults living with type 1 diabetes.

**Materials and Methods:**

This descriptive and correlational study was conducted in three hospitals of Turkey between July 4, 2022, and March 1, 2023. The study sample consisted of 150 persons with type 1 diabetes aged 12–22 who attended these hospitals. Data were collected using the “Patient Information Form,” “Diabetes Technology Questionnaire (DTQ),” and “Type 1 Diabetes Stigma Assessment Scale (DSAS-1).”

**Results:**

The disease-related characteristics of the participants were examined. It was found that 92% used an insulin pen, 90.6% used a glucometer for glucose measurement, 57.4% measured glucose in public places such as buses and cafes, and 33.8% experienced discrimination in public while measuring glucose. Additionally, 52.7% experienced hyperglycemia, 44.7% experienced hypoglycemia, and 10.7% had a chronic disease. In multiple regression analyses, higher perceived stigma (DSAS-1 total; standardized *β* =  − 0.64, *p* < .001) and the number of blood glucose measurements per day (*β* =  − 0.225, *p* = .029) were independently associated with lower satisfaction with diabetes technologies (DTQ), after adjusting for covariates.

**Conclusions:**

As DTQ scores increased, the DSAS-1 total scores decreased. Improving access to diabetes technologies for individuals with type 1 diabetes should be prioritized. Additionally, randomized controlled studies could be conducted to further explore the impact of diabetes technologies on perceived stigma in people with diabetes.

## Introduction

Type 1 diabetes is a lifelong disease with high mortality and morbidity rates, and it can lead to various complications. According to data published by the International Diabetes Federation (IDF), an estimated 8.8 million people worldwide have type 1 diabetes, including 1.2 million children aged 0–19 years (IDF, 2021). Additionally, it is reported that 25,759 individuals aged 0–19 years in Turkey have type 1 diabetes (IDF, 2021). Type 1 diabetes is a lifelong disease associated with high rates of mortality and morbidity and can lead to various complications (ElSayed et al., 2023). As a result, lifelong medical nutrition therapy, insulin therapy, exercise management, and continuous diabetes education are essential for individuals with type 1 diabetes. With proper diabetes management, complications can be prevented, and reductions in hospitalizations and mortality rates can be achieved (ElSayed et al., 2023).

People with type 1 diabetes (PwT1D) must rely on insulin injections, blood glucose monitoring, and other diabetes technologies throughout their lives to manage the disease. However, PwT1D frequently experience stigma due to being diagnosed at a young age, encountering frequent challenges with their peers (particularly in the adolescent age group), and needing to use diabetes technologies (such as insulin injections, blood glucose monitoring, or other diabetes devices) in school and social settings (Liu et al., 2017; Mutlu, 2019).

The concept of stigma refers to the situation where an individual is blamed or excluded by society simply because they are perceived as different from what is considered normal in that society (Uz & Kaya, 2018). Stigma can negatively affect individuals in various aspects, including socially, emotionally, and psychologically (Beverly et al., 2019; Uz & Kaya, 2018). Individuals with chronic illnesses often experience stigma due to various factors, including disease progression, functional limitations and disability, mandatory lifestyle changes, reduced participation in social activities, low levels of self-efficacy, and insufficient social support (Ji et al., 2024; Brown, 2015; Ghajarzadeh et al., 2024; Toumi et al., 2024; Sajatovic et al., 2024).

PwT1D are likely to be constantly stigmatized because individuals constituting the society have insufficient knowledge about type 1 diabetes, because they hold the patient responsible for the disease, because they have false thoughts that type 1 diabetes is contagious and because they have prejudiced thoughts that these patients with diabetes have inadequacies/deficiencies (Liu et al., 2017; Abdoli, Hardy & Hall, 2017). Self-esteem and glycemic control of individuals who experience stigma is negatively affected; their body image deteriorates; they experience psychological problems such as depression, anxiety and anger (Beverly et al., 2019; Ortiz-Domenech & Cumba-Avilés, 2021). In this context, individuals with type 1 diabetes (PwT1D) are particularly vulnerable to stigmatization, especially due to the ongoing need for treatment and the responsibility associated with managing the disease (Liu et al., 2017; Abdoli, Hardy & Hall, 2017; Mutlu, 2019; Embick, Jackson & Stewart, 2024). Studies have shown that individuals diagnosed with type 1 diabetes experience stigma at rates ranging from 60% to 93% (Abdoli, Abazari & Mardanian, 2013; Browne et al., 2014; Brazeau et al., 2018; Ortiz-Domenech & Cumba-Avilés, 2021; Eitel et al., 2024; Holmes-Truscott et al., 2025). Individuals who experience stigma may avoid administering insulin injections in public, resulting in delays in their treatment. As a consequence, those who are hesitant to follow proper diabetes self-management practices often experience poor diabetes control, which increases the risk of diabetes-related complications (Uz & Kaya, 2018; Sürücü, Durmaz & Turan, 2020; Eitel et al., 2024).

The effective management of diabetes depends on individuals’ satisfaction with the treatment and care services they receive (Can, 2019). However, defining the concept of satisfaction is challenging, as individual satisfaction is influenced by numerous variables. These variables include the quality of services provided by the hospital, the appropriateness of the treatment, the patient’s active participation in the treatment decision-making process, the attitudes and behaviors of healthcare personnel, the physical conditions of the hospital, and the overall quality of care (Taşlıyan & Gök, 2012; Charleer et al., 2020). Satisfaction is generally defined as a positive emotional evaluation arising from a situation in which an individual’s expectations are met, thereby leading to a sense of happiness (Taşlıyan & Gök, 2012).

People with diabetes (PwD) should be satisfied with their treatment, care, and the diabetes technologies they use, as higher satisfaction is associated with improved diabetes management, enhanced quality of life, and the prevention of complications (Can, 2019; Charleer et al., 2020). Several studies have examined satisfaction with the use of diabetes technologies in PwD (Eren & Tarım, 2017; Orhan & Bahçecik, 2018; Gülen, Korkusuz & Korkusuz, 2022; Alsahli et al., 2024; Franceschi et al., 2024). Some studies report that PwD and their families are satisfied with diabetes technologies and recommend them due to improvements in metabolic control, quality of life, and a reduction in complications (Özkaya, 2017; Gülen, Korkusuz & Korkusuz, 2022). On the other hand, some studies have reported that factors such as the high cost of devices, negative changes in body image, increased technological dependence and self-stigma, difficulties in adapting to the technology, and privacy concerns reduce individuals’ satisfaction with these technologies (Özkaya, 2017; Büyükkaya & Dervişoğlu, 2021; Feliziani, Chios & Pozzilli, 2024).

Although the literature includes numerous studies examining stigma and satisfaction with diabetes technologies separately, no study has yet evaluated the relationship between these two concepts. However, a systematic review by Natale et al. (2023), synthesizing qualitative findings from studies with PwD, reported that the use of diabetes technologies may increase stigma and lead to dissatisfaction. This stigma was associated with factors such as the bulky appearance of the device, the disturbing noise it produces, and the embarrassment experienced when others inquire about the device (Natale et al., 2023). Therefore, the aim of this cross-sectional study is to address this gap in knowledge by examining the association between perceived stigma and satisfaction with diabetes technologies among adolescents and young adults living with type 1 diabetes, thereby offering a novel contribution to the existing literature.

Research questions:

• Is there a relationship between satisfaction with diabetes technology use and stigma?
• Is there a relationship between individuals’ disease-specific characteristics and diabetes technology scores?

## Materials & Methods

### Study design, setting, and participants

Using a descriptive and correlational research design, this study was conducted in three hospitals of Turkey (located in Batman and Diyarbakır) between July 4, 2022, and March 1, 2023. A descriptive and correlational research design was chosen to examine the relationship between stigma and diabetes technologies. Due to the limited number of individuals with type 1 diabetes aged 12–22 years and the fact that this patient group is followed only at specific centers, three tertiary-level training and research hospitals located in the provinces of Batman and Diyarbakır were selected as the study sites. All patients registered at diabetes education centers and hospital outpatient clinics who met the inclusion criteria and provided informed consent were recruited for the study.

The sample of the study consisted of 150 people with type 1 diabetes (PwT1D), aged between 12 and 22, who visited these hospitals and were selected using a non-probability sampling method.

### The criteria for inclusion in and exclusion from the study

Individuals diagnosed with type 1 diabetes at least six months prior to the study, who used diabetes technologies such as insulin injection, glucometer, insulin pen, or insulin pump, and who were able to speak and understand Turkish well enough to communicate, were included in the research sample. Individuals with other specific types of diabetes, such as type 2 diabetes and gestational diabetes or those who had health conditions potentially contributing to stigma (such as schizophrenia, depression, cancer, long-term treatments like dialysis, infectious diseases, or physical limitations) were excluded from the study.

Using the G*Power 3.0.10 software, a post hoc linear multiple regression analysis was conducted. With a sample size of 150, an effect size of 0.2, 23 tested predictors, and a significance level of 0.05, the statistical power of the study was calculated as 0.88 (Faul et al., 2007).

### Data collection

Due to the ongoing pandemic during data collection and the difficulty in reaching individuals aged 12–22 at the hospital, contact information of patients who had visited the diabetes education centers and outpatient clinics was obtained from the hospital. Online interviews were then conducted *via* WhatsApp with those who agreed to participate. Participants completed the Patient Information Form, DTQ, and DSAS-1 as self-administered online questionnaires, providing their responses directly through the online platform. The completion of the questionnaires took approximately 15–20 min.

#### Data collection tools

##### Patient information form.

This form was prepared by the researchers based on a review of the relevant literature (Taşlıyan & Gök, 2012; Liu et al., 2017; Uz & Kaya, 2018). This form consists of a total of 20 items addressing the socio-demographic characteristics of PwT1D—including gender, age, marital status, cohabitation status, place of residence, educational level and income level—as well as detailed aspects related to their illness, such as exercise habits (such as brisk walking, running, swimming or resistance exercises aimed at increasing muscle strength), insulin administration method, blood glucose monitoring method and location, experiences of discrimination during blood glucose measurement in public places, presence of hyperglycemia (fasting plasma glucose levels exceeding 140 mg/dL and postprandial (post-meal) plasma glucose levels exceeding 180 mg/dL) and hypoglycemia complications (Plasma glucose levels below 70 mg/dL), comorbid chronic diseases (*e.g.*, chronic obstructive pulmonary disease, asthma, hypertension, thyroid disorders, osteoporosis, cancer, myocardial infarction), hemoglobin A1C (HbA1C) levels, duration of diabetes, daily frequency of blood glucose measurements, and duration of diabetes technology usage. In addition, HbA1c measurements were collected from patients *via* self-reports and verification through medical record review.

Continuous glucose monitoring (CGM) devices automatically record interstitial glucose values approximately every 5 min; therefore, these automated readings were not included in the calculation of “daily blood glucose measurement frequency”, as they do not reflect patient-initiated measurements. For participants using CGM, only additional fingerstick (capillary) measurements performed for confirmation, calibration, or as needed were counted as daily blood glucose measurements. Consequently, the variable “number of blood glucose measurements per day” was defined based on patient-initiated, invasive fingerstick tests, regardless of CGM use.

##### Diabetes technology questionnaire.

The scale, originally developed by Prof. Dr. Tim Wysocki as the ‘Continuous Glucose Monitoring Satisfaction Scale,’ was revised in 2014 to become the ‘Diabetes Technology Scale’ (Can, 2019). The Diabetes Technology Questionnaire was used to measure the satisfaction of children and adolescents with the diabetes technologies used in the management of type 1 diabetes, and the Turkish validity and reliability study of the scale was conducted by Ecem Can. The Diabetes Technology Questionnaire (DTQ) consists of a total of 30 items and has two versions: (1) the initial/basic questionnaire, which evaluates the patient’s experience and satisfaction with their ‘current’ diabetes technology package, and (2) the follow-up questionnaire, designed to assess the ‘current’ effect/satisfaction as well as the ‘change’ in effect/satisfaction compared to pre-study levels. The scale is a five-point Likert-type scale (Can, 2019). Responses, such as “Very much” and “Not at all”, were assigned numerical values from one to five, and participants selected one of these values. The mean item score of the scale was calculated by summing the scores for each item answered and dividing by the number of items answered. For example, if 20 questions were answered, the total score was divided by 20. After conducting the reliability analysis, Cronbach’s alpha coefficient was found to be 0.89, indicating that the scale is reliable. A higher total score on the scale indicates greater satisfaction with the use of diabetes technology (Can, 2019).

##### Type 1 Diabetes Stigma Assessment Scale (DSAS-1).

The Turkish validity and reliability study of the scale, developed by Browne, Ventura, Speight, and Mosely in Australia in 2017, was conducted by Mutlu (2019). The scale consisted of 19 items in total, with three sub-dimensions: being treated differently (3, 6, 8, 12, 15, and 19 items), blame judgment (1, 4, 9, 11, 14, and 17 items), and identity concerns (2, 5, 7, 10, 13, 16, and 18 items). Each subscale evaluates a different dimension of the stigma experience, while the total score of the scale reflects the overall level of stigma. It offered five response options: ‘strongly disagree,’ ‘disagree,’ ‘neutral,’ ‘agree,’ and ‘strongly agree.’ Based on the responses, it was determined whether the individual experienced stigmatization. The Cronbach’s alpha coefficient for the entire scale was found to be 0.89, indicating that the scale is reliable. The minimum possible score on the scale was 19, while the maximum possible score was 95. As the score increased, the level of stigmatization felt by the individual also increased (Mutlu, 2019). Since this study aimed to examine the overall level of stigma in individuals with type 1 diabetes, analyses were conducted using the total scale score.

### Data analysis

The data collected were analyzed using the SPSS software (IBM SPSS Statistics 26). Descriptive statistics, including percentages and mean ± standard deviation (SD), were used to present the data. In this study, multiple linear regression analysis was conducted using the Enter (forced entry) method, in which all independent variables were entered into the model simultaneously. Prior to the analysis, the assumptions of regression were tested. The linear relationship between the dependent and independent variables was examined using scatterplots, which indicated that the assumption of linearity was met. The independence of residuals was assessed with the Durbin–Watson statistic, and a value of 2.03 indicated the absence of autocorrelation. The assumption of homoscedasticity was verified by the random distribution of standardized residuals against predicted values. The normality of residuals was evaluated using the Normal P–P Plot and histogram, both of which showed that the data points clustered around the reference line without significant deviations. Multicollinearity was assessed through Variance Inflation Factor (VIF) and tolerance values; all variables had VIF values below 5 and tolerance values above 0.10, indicating no multicollinearity. Influential outliers were examined using Cook’s Distance, with all values found to be below 1. These findings confirm that all key assumptions of regression analysis were satisfied. A statistical significance level of *p* < 0.05 was accepted for all tests.

### Ethical aspects of the research

Ethical approval for this study was obtained from the Dicle University Social and Human Sciences Ethics Committee (Ethics Committee Decision: June 17, 2022, No. 175; Rectorate Approval: June 27, 2022, No. 313199) (File 1). For participants aged 18 years and older, informed consent was obtained by having them read and sign the Informed Consent Form (File 2). For participants under 18 years of age, parental consent was obtained through the completion of the Parental Consent Form (File 3). Following parental consent, the questionnaires were administered to minors by the researcher, and the responses were recorded. Permissions were also obtained from the relevant institutions to conduct the study. Additionally, authorization was secured from the authors who conducted the validity and reliability studies of the “Diabetes Technology Questionnaire” and the “Type 1 Diabetes Questionnaire” for the use of these scales in the research (Files 4 and 5).

## Results

According to the results, the socio-demographic characteristics of the participants were as follows: 42.7% were aged ≥ 20 years, 54.0% were female, 96% were single, 97.3% lived with their families, 93.3% lived in the city, 42.7% were high school graduates and 86.7% had no income (Table 1).

When examining the disease-related characteristics, it was found that 76.7% of participants exercised, 92% used an insulin pen, 90.6% used a glucometer for glucose measurement, 57.4% measured glucose in public places such as buses and cafes, 33.8% experienced discrimination while measuring glucose in public places, 52.7% experienced hyperglycemia, 44.7% experienced hypoglycemia, and 10.7% had a chronic disease (Table 2).

Furthermore, the mean HbA1c score was 8.13 (± 1.83), the mean duration of diabetes diagnosis was 6.36 years (± 4.45), the mean number of blood glucose measurements per day was 4.85 (± 2.91), and the mean duration of diabetes technology use (including glucometers and continuous glucose monitoring) was 6.36 years (± 4.45). The mean total diabetes technology satisfaction score was 3.44 (± .92), and the mean total type 1 diabetes stigma score was 46.76 (± 18.17) (Table 2).

**Table 1:** Distribution of demographic characteristics of ındividuals with type 1 diabetes.

**Variable (*N* = 150)**	**n/ Mean**(±**)**	**% (min-max)**
**Gender**		
Female	81	54.0
Male	69	46.0
**Age classes** [Mean(±). → 17.87 ± 3.42(year)]		
≤ 14	38	25.3
15–19	48	32.0
≥ 20	64	42.7
**Marital Status**		
Married	6	4.0
Single	144	96.0
**People who they lived with**		
Alone	4	2.7
With parents	146	97.3
**Place of accommodation**		
Village	10	6.7
City	140	93.3
**Education**		
Illiterate	18	12.0
Elementary School	29	19.3
High School	64	42.7
University	39	26.0
**Level of income**		
No income	130	86.7
500–2.500 TL	15	10.0
2.501–5.000 TL	5	3.3

**Table 2:** Distribution of individuals with type 1 diabetes according to disease-related characteristics and other variables.

**Variable (*N* = 150)**	**n/Mean** (±)	**% (min–max)**
**Doing exercise** ^*^		
Yes	115	76.7
No	35	23.3
**Insulin administration**		
Insulin pen	138	92.0
Insulin pump	11	8.0
**Way of measuring blood glucose**		
Glucometer device	136	90.6
Continuous glucose monitoring devices (CGM)	14	9.3
**Place of blood glucose measurement**		
Home	64	42.6
Open environments like bus, café	86	57.4
**Thinking of being exposed to discrimination in open environments while measured glucose in public places**		
Yes	29	33.8
No	57	66.2
**Hyperglycemia**		
Yes	79	52.7
No	71	47.3
**Hypoglycemia**		
Yes	67	44.7
No	83	55.3
**Having chronic disease**		
Yes	16	10.7
No	134	89.3
HbA1c	8.13 ± 1.83	(5.1–13.00)
Diabetes Duration (Year)	6.36 ± 4.45	(0.6–20.0)
Number of blood glucose measurements per day (times/day)	4.85 ± 2.91	(0–20.00)
Diabetes Technology (Glucometer device and CGM) usage time (Year)	6.36 ± 4.45	(0.6–20.0)
Total Diabetes Technologies Satisfaction Score	3.44 ± .92	(1.0–5.0)
Total Type 1 Diabetes Stigma Score	46.76 ± 18.17	(19.0–94.0)

**Notes.**

* It refers to aerobic exercises (such as brisk walking, running, swimming) or resistance exercises.

In the multiple linear regression analysis conducted using the enter method, factors predicting satisfaction with diabetes technologies among individuals with type 1 diabetes were examined. The model was found to be statistically significant (*F* = 3.693, *p* < .001) and explained 52.3% of the variance in satisfaction scores (Adj. *R*^2^ = .523). In multiple regression analyses, higher perceived stigma (DSAS-1 total; standardized *β* = −0.64, *p* < .001) and the number of blood glucose measurements per day (*β* = −0.225, *p* = .029) were independently associated with lower satisfaction with diabetes technologies (DTQ), after adjusting for covariates. No significant associations were observed between satisfaction and other variables, including age, gender, marital status, education level, income level, duration of diabetes, engagement in regular exercise, method of insulin administration, duration of diabetes technology use, method and setting of blood glucose measurement, perception of discrimination, presence of hyperglycemia or hypoglycemia, presence of chronic disease, and HbA1c levels (Table 3).

**Table 3:** Predictors of diabetes technologies satisfaction in patients with type 1 diabetes.

	**Diabetes Technologies Satisfaction**			
**Variables** ^**^	**Beta**	**SE**	**t**	***p***
Age (Year)	−0.206	0.050	−1.251	0.216
Gender (Female)	−0.064	0.186	−0.618	0.539
Marital Status (Married)	−0.035	0.473	−0.267	0.791
People who they lived with (Alone)	0.089	0.377	0.944	0.349
Place of accommodation (Village)	−0.127	0.461	−0.992	0.326
Education (Elementary School)	0.004	0.451	0.031	0.976
Education (High School)	0.178	0.316	1.011	0.317
Education (University)	0.267	0.378	1.318	0.193
Level of income (500–2.500 TL)	0.112	0.334	0.930	0.357
Level of income (2.501–5.000 TL)	−0.002	0.747	−0.025	0.980
**Total Type 1 Diabetes Stigma Score**	**−0**.**644**	**0**.**005**	**−6**.**094**	**0**.**000**
Diabetes Duration (Year)	−0.027	0.092	−0.064	0.949
Doing exercise (Yes)	−0.002	0.243	−0.020	0.984
Insulin administration method (pen)	0.114	0.474	1.105	0.274
Diabetes Technology (Glucometerdevice and CGM) usage time (Year)	0.234	0.089	0.570	0.571
Way of measuring blood glucose	−0.002	0.240	−0.026	0.979
**Number of blood glucose measurements per day (times/day)**	**−0**.**225**	**0**.**034**	**−2**.**251**	**0**.**029**
Place of blood glucose measurement (open environment like school or hospital)	−0.182	0.789	−1.290	0.203
Thinking of being exposed to discrimination in open environments (Yes)	−0.143	0.228	−1.187	0.241
Hyperglycemia (Yes)	0.029	0.183	0.293	0.771
Hypoglycemia (No)	0.162	0.177	1.642	0.107
Having chronic disease (Yes)	0.140	0.278	1.554	0.126
HbA1C	0.022	0.047	0.245	0.808
	^*^Model R^2^ : .669, Adjusted R^2^ : .523, F: 3.693, *p* < .001			

**Notes.**

* Multiple linear regression analysis was conducted using the Enter (forced entry) method.

** In the present study, gender (female = 1, male = 0), marital status (married), living alone (yes), place of residence (village), exercise status (yes = 1, no = 0), insulin administration method (pen), place of blood glucose measurement (open environments such as school or hospital), perception of being exposed to discrimination (yes), history of hyperglycemia (yes), history of hypoglycemia (no), and presence of chronic disease (yes) were defined as dummy variables.

Additionally, for the education variable, the category “illiterate” was designated as the reference value, while for the economic status variable, the category “no income” was set as the reference value. For education status, elementary school, high school, and university; and for economic status, “500–2,500 TL” and “2,501–5,000 TL” were included in the model as dummy variables.

*** *p* < 0.05.

## Discussion

In this study, we examined the relationships between satisfaction with diabetes technology use and stigma levels, as well as the associations between disease-specific characteristics and diabetes technology scores among adolescents and young adults living with type 1 diabetes. The findings were then discussed within the context of the existing literature.

In this study, the total DSAS-1 score was found to be 46.76 (maximum score: 94), indicating that the perceived level of stigma was moderate. This result is consistent with studies in the literature (Himmelstein & Puhl, 2021; Ingram, Ohan & Bebbington, 2022; Sińska et al., 2023). PwD frequently experience stigmatization due to misconceptions in society, such as beliefs that malnutrition or obesity cause diabetes, that individuals are entirely responsible for the development of the disease, that diabetes is contagious, and that insülin use resembles drug abuse (Liu et al., 2017; Abdoli, Hardy & Hall, 2017; Mutlu, 2019). In addition, individuals with type 1 diabetes are also exposed to stigma due to factors such as the lack of public awareness about type 1 diabetes, the difficulties individuals face while managing the disease, and the complications they experience (Liu et al., 2017; Mutlu, 2019; Abdoli, Hardy & Hall, 2017; Embick, Jackson & Stewart, 2024). The lack of public awareness about type 1 diabetes may have contributed to the stigmatization of PwT1D.

The Diabetes Technology Questionnaire (DTQ) score of 3.44 was considered high, indicating that most PwT1D were satisfied with diabetes technologies. This finding is supported by previous studies, where the majority of participants reported satisfaction with diabetes technologies (Eren & Tarım, 2017; Grando et al., 2019; Charleer et al., 2020; Polat & Avdal, 2021; Pinsker et al., 2021; Gülen, Korkusuz & Korkusuz, 2022; Alsahli et al., 2024; Franceschi et al., 2024). Although modern diabetes technologies, such as insulin pumps and continuous glucose monitoring (CGM) systems are available in Turkey, their use remains limited—particularly among children and adolescents—primarily due to high cost, limited insurance coverage, and lack of awareness (Gülen, Korkusuz & Korkusuz, 2023; Kardaş & Gürol, 2022). These contextual details help situate our findings, as limited uptake may influence patients’ perceptions of diabetes management and satisfaction with technology. Modern diabetes technologies, such as insulin pumps and CGM devices, allow for more precise insulin dosing, increased greater daily-life flexibility, enhanced quality of life, improved metabolic control, and reduced diabetes-related complications (Gülen, Korkusuz & Korkusuz, 2023; Eren & Tarım, 2017; Kardaş & Gürol, 2022; Buğrul et al., 2016; Çamurdan et al., 2008). Conversely, traditional methods such as fingerstick blood glucose monitoring have been widely used for many years and are considered reliable, offering individuals a sense of security through the ability to confirm glucose levels when needed. While traditional methods remain essential in emergencies, modern technologies provide significant advantages for daily management (Eren & Tarım, 2017; Gonzales, Mobashsher & Abbosh, 2019; Charleer et al., 2020; Natale et al., 2023; Bolla & Priefer, 2020; Gülen, Korkusuz & Korkusuz, 2022; Ingram, Ohan & Bebbington, 2022; Buğrul et al., 2016). The reliability and practicality of these technologies likely contribute to the high satisfaction levels observed in PwT1D, which may in turn reduce perceived stigma (DSAS-1) and improve psychosocial well-being.

Stigmatization was identified as a significant predictor of diabetes technology satisfaction among PwT1D. The results demonstrated that higher diabetes technology satisfaction scores were associated with lower DSAS-1 scores. In the existing literature, there is no research examining the relationship between diabetes technology satisfaction and stigma in PwT1D. Looking at the relationship between stigma and diabetes technologies, a systematic review compiling qualitative studies found that stigma can affect technology use, personal care activities, help-seeking behavior, and even education and employment status for PwT1D (Embick, Jackson & Stewart, 2024). Consistent with our findings, there appears to be a negative relationship between diabetes technology use and the perception of diabetes stigma. An increase in perceived stigma may have led to a decrease in diabetes technology satisfaction in PwD. Diabetes patients who administer treatment in public settings using traditional methods—particularly insulin injections and blood glucose measurements—are often mistakenly perceived by bystanders as engaging in illicit drug use, which may expose them to stigma and prejudice (Sürücü, Durmaz & Turan, 2020; Mehmet, Hussey & Ibrahim, 2015; Liu et al., 2017; Mutlu, 2019). Evidence from both qualitative and quantitative research indicates that such stigma can lead diabetes patients to postpone treatment, avoid administering insulin in public, and consequently experience disruptions in diabetes management (Mehmet, Hussey & Ibrahim, 2015; Liu et al., 2017; Mutlu, 2019). This process has additional negative repercussions on psychosocial well-being; individuals experiencing stigma have been reported to exhibit higher rates of complications, depression, negative body image, and low self-esteem (Liu et al., 2017; Mutlu, 2019; Can, 2019; Beverly et al., 2019; Artuvan & Yurtsever, 2020; Ortiz-Domenech & Cumba-Avilés, 2021; Andersen, Varga & Folker, 2022). Advanced technologies such as CGM systems and insulin pumps, while improving clinical outcomes, may also elicit concerns about visual and auditory stigma due to their noticeable size and the sounds they emit during operation, as demonstrated in qualitative studies (Kardaş & Gürol, 2022; Natale et al., 2023; Shrestha et al., 2024). Consequently, some users prefer to conceal their devices or operate them in environments where they are less likely to be noticed (Natale et al., 2023; Shrestha et al., 2024). Conversely, other diabetes patients report that CGM use enhances their self-confidence and facilitates greater participation in activities such as sports, travel, and work, by eliminating the need for frequent manual blood glucose monitoring (Natale et al., 2023). From a cultural perspective, in Turkey, where many individuals—including diabetes patients—tend to dress modestly, the visibility of such devices may be less socially problematic, potentially fostering greater social integration and reducing perceptions of stigma. Given that the present study’s sample primarily comprised adolescents and young adults, it is plausible that the use of modern diabetes technologies in this group enhanced self-confidence and reduced perceived stigma. A systematic review of qualitative research on the relationship between diabetes technologies and stigma found that stigma can influence the technology use, self-care activities, help-seeking behaviors, and even educational and employment outcomes of PwT1D (Embick, Jackson & Stewart, 2024). It may not be the use of diabetes technology per se that is associated with perceived stigma, but rather that higher treatment satisfaction (DTQ) is negatively correlated with perceived stigma (DSAS-1).

In this study, the number of blood glucose measurements per day was found to be a significant predictor of diabetes technology satisfaction in PwT1D. The results showed that as the number of daily blood glucose measurements increased, satisfaction with diabetes technologies decreased. Since diabetes is a lifelong disease, continuous blood glucose measurement is necessary. These measurements are done using invasive and minimally invasive devices (Göktaş, Çankaya & Ermeydan, 2022). Glucometers require more invasive procedures (on average 1800 times a year) than other technologies like CGMs, insulin pumps, or Flash Monitoring, and they may cause pain, finger tissue damage, an increased risk of infection, and reduced quality of life for users (Nakayama et al., 2008; Kocher, Tshiananga & Koubek, 2009; Zhang et al., 2011; Małachowska et al., 2016; Can, 2019; Gonzales, Mobashsher & Abbosh, 2019; Charleer et al., 2020; Bolla & Priefer, 2020; Göktaş, Çankaya & Ermeydan, 2022). Several factors may explain why increased frequency of daily blood glucose measurements is associated with lower satisfaction with diabetes technologies. First, the traditional glucometers and the continued reliance on fingerstick measurements (even among CGM users) can lead to pain and tissue damage. Second, individuals who feel uncomfortable measuring their blood glucose levels in public settings may become increasingly self-conscious as the frequency of these measurements rises, which can amplify their perception of stigma and social pressure.

## Conclusion

In our study, it was observed that higher Diabetes Technology Survey scores were associated with lower total DSAS-1 scores. Further research is recommended to examine the impact of cultural differences on reducing perceived stigma and enhancing satisfaction with diabetes technologies. Based on our findings, randomized controlled trials could also be conducted to investigate the effects of diabetes technologies on perceived stigma among people with diabetes (PwD). Moreover, additional research aimed at reducing stigma in this population is warranted, and the findings from such studies should be supported by further investigations exploring their potential to improve satisfaction with diabetes technologies.

This study identified that the perceived stigma, as measured by the DSAS-1, was at a “moderate” level. There is a critical need for a multidimensional and multilayered approach to reduce stigma among individuals living with type 1 diabetes mellitus. Such an approach should include increasing public awareness and understanding of the disease, encouraging healthcare professionals to adopt holistic care models rather than relying solely on biomedical methods, and guiding individuals in developing effective coping strategies. Addressing cases of discrimination through collaboration between diabetes specialists, support groups, or both is crucial for gaining social and political support. Furthermore, diabetes care teams should remain vigilant for signs of discrimination and implement preventive measures. Healthcare providers should also be aware of the cultural and religious beliefs of families and tailor care plans accordingly. To minimize high levels of stigma, governmental and media policies should be leveraged to raise community awareness; school-based programs should be expanded; social interaction opportunities should be promoted through group activities; continuous education should be provided by healthcare professionals; and individuals should be encouraged to take an active role in their diabetes management.

Our study also revealed that participants reported a “high” level of satisfaction with diabetes technologies. However, satisfaction decreased as the number of daily blood glucose measurements increased. Therefore, expanding access to diabetes technologies that can reduce the frequency of daily blood glucose testing is recommended. To improve accessibility—particularly for newer options such as CGM systems and insulin pumps—greater governmental support should be provided. Additionally, individuals should be further educated regarding the use of diabetes technologies. Public awareness of diabetes technology use could be enhanced through congresses, symposiums, educational programs, and media initiatives.

It is noteworthy that the majority of participants primarily used traditional diabetes technologies (insulin pens and glucometers), while the number of individuals using advanced technologies such as insulin pumps and CGM devices was limited. Therefore, future research should aim to include larger and more diverse samples from different regions, incorporating individuals with higher levels of diabetes technology usage.

## Limitations of the Study

This study was conducted exclusively with individuals aged 12–22 years diagnosed with type 1 diabetes, recruited from three hospitals located in two provinces of Turkey (Batman and Diyarbakır). Due to the limited number of patients within this age group and the challenges encountered in reaching an appropriate sample, a non-probability sampling method was employed. This may limit the generalizability of the study findings. Furthermore, as the data in this study were collected through self-report, potential biases may have influenced the accuracy of the measurements. These include participants’ tendency to provide socially desirable responses (social desirability bias), difficulties in recalling past information (recall bias) and possible limitations in the accuracy of self-reported data. In addition, the cross-sectional design of the study prevents determination of causal relationships between variables and does not allow tracking of changes over time. Therefore, the findings reflect only the conditions present during the period in which the data were collected, and different results may be obtained in other time frames or contexts, which should be considered a limitation of the study.

##  Supplemental Information

10.7717/peerj.20884/supp-1 Supplemental Information 1 Data

10.7717/peerj.20884/supp-2 Supplemental Information 2 STROBE checklist

## Additional Information and Declarations

### Data Availability

The following information was supplied regarding data availability: The raw data is available in the Supplemental File.
